# Molecular Mechanisms of Cardiac Amyloidosis

**DOI:** 10.3390/ijms23010025

**Published:** 2021-12-21

**Authors:** Yukihiro Saito, Kazufumi Nakamura, Hiroshi Ito

**Affiliations:** 1 Department of Cardiovascular Medicine, Okayama University Hospital, Okayama 700-8558, Japan; p5438a3l@s.okayama-u.ac.jp; 2 Department of Cardiovascular Medicine, Okayama University Graduate School of Medicine, Dentistry and Pharmaceutical Sciences, Okayama 700-8558, Japan; itomd@md.okayama-u.ac.jp

**Keywords:** amyloid, cytotoxicity, transthyretin, immunoglobulin light chain

## Abstract

Cardiac involvement has a profound effect on the prognosis of patients with systemic amyloidosis. Therapeutic methods for suppressing the production of causative proteins have been developed for ATTR amyloidosis and AL amyloidosis, which show cardiac involvement, and the prognosis has been improved. However, a method for removing deposited amyloid has not been established. Methods for reducing cytotoxicity caused by amyloid deposition and amyloid precursor protein to protect cardiovascular cells are also needed. In this review, we outline the molecular mechanisms and treatments of cardiac amyloidosis.

## 1. Introduction

Amyloidosis is a general term for diseases in which misfolded proteins form amyloid fibrils that are rich in a unique β-sheet structure and the amyloid fibrils are deposited in various organs throughout the body and cause organ dysfunction [1]. In ATTR amyloidosis and AL amyloidosis, amyloid deposition in the heart is a major cause of poor prognosis [2,3,4,5]. The causative proteins of these amyloidosis forms have been identified and the mechanisms of amyloid aggregation have been elucidated, and promising treatments have emerged with the evolution of biotechnology [5,6,7,8,9,10]. However, there are still unclear points, such as the cardiotropic mechanisms and amyloid removal mechanisms, and it is thought that elucidation of these points will lead to better treatment. In this review, we describe the molecular mechanisms and treatments of cardiac amyloidosis.

## 2. Cardiac Amyloidosis

Amyloid is a water-insoluble fibrillar protein that is rich in a β-sheet structure. Amyloid precursor protein causes folding disorders and it polymerizes, accumulates and aggregates as insoluble fibers [1]. Moreover, several other proteins, such as heparan sulphate proteoglycan and serum amyloid P-component, are always co-deposited with amyloid fibrils [11]. Recent development of proteomic analyses have revealed proteins included in amyloid deposits [12,13].

The pathological condition that causes morphological and functional abnormalities by deposition of amyloid fibers in the interstitium of the heart is called cardiac amyloidosis. The major types of cardiac amyloidosis are ATTR amyloidosis and AL amyloidosis (Table 1) [12]. There are many similarities in clinical symptoms and laboratory findings among disease types. Larsen et al. reported differences in histopathological findings between ATTR amyloidosis and AL amyloidosis and they reported that the extent and distribution of cardiac amyloid deposits correlate with amyloid type, suggesting fundamental differences in the pathobiology of deposition [14]. The pathological condition is mainly due to cardiac hypertrophy and diastolic dysfunction. Compression of microvessels by the hypertrophic myocardium and amyloid deposition in microcoronary arteries within the myocardial wall may cause myocardial ischemia [15,16,17]. Impaired contraction, impaired atrioventricular conduction, atrial fibrillation and lethal arrhythmia are also seen in cardiac amyloidosis. Organ damage and prognosis depend not only on the cumulative deposition of amyloid fibrils but also on the type of fibrils deposited. This has been demonstrated by the worse prognosis for cardiac amyloidosis in AL fibrils than in ATTR fibrils, despite high cumulative fibril deposition in ATTR amyloidosis [18]. In recent years, effective treatments for ATTR amyloidosis and AL amyloidosis have been developed.

## 3. Molecular Mechanisms of Amyloidosis

Displacement of normal parenchymal tissue with amyloid deposits is not sufficient to fully explain the organ dysfunction associated with both AL and ATTR cardiomyopathy. Nerve biopsy or cardiac autopsy specimens from patients with ATTR amyloidosis and patients with AL amyloidosis showed atrophy and degeneration of cells surrounding amyloid fibrils [19]. The toxicity of oligomers may also be involved in the mechanisms of tissue damage. Moreover, extracellular misfolded and aggregated proteins trigger intracellular signal cascades associated with inflammation, oxidative stress and matrix remodeling [20]. We explain the molecular mechanisms of ATTR amyloidosis and AL amyloidosis, which mainly cause cardiac amyloidosis.

### 3.1. ATTR Amyloidosis

#### 3.1.1. Wild-Type ATTR Amyloidosis

Wild-type ATTR (ATTRwt) amyloidosis mainly impairs the heart, tendon and ligament tissues (hands, root canal, ligamentum flavum, etc.), kidneys, thyroid, peripheral nerves and lungs. It is common in men over the age of 60 years, and aging might be involved in its onset.

In 1990, it was reported that the causative protein of ATTRwt amyloidosis was transthyretin (TTR) [21]. TTR is a 55-kDa homotetrameric protein produced in the liver, choroid plexus and retinal pigment epithelium and plays a role in transporting thyroxine (T_4_) and retinol-binding protein in serum and cerebrospinal fluid [22,23,24]. TTR exists stably in the blood by forming a tetramer, but with aging, the tetramer becomes unstable and dissociates into monomers that misfold and become substrate for amyloid fibrils formation [25]. The stability of the TTR tetramer is the rate-limiting step for aggregation and amyloid fibril formation [26,27].

The native TTR monomer is a 127-amino-acid protein with a short α-helix motif and eight β-strand chains. These eight β-strand chains form two four-β-stranded anti-parallel sheets, DAGH and CBEF β-sheets, respectively [24,28]. Yang et al. showed various transient structural states of TTR by using nuclear magnetic resonance experimental data analysis and molecular dynamics studies [29]. Structural deformation of the DAGH β-sheet and the AB loop regions may correlate with the development of the aggregation-prone conformational states of TTR.

The mechanism by which tetramers tend to dissociate with aging is still unclear, but it is speculated that it may be due to post-translational biochemical changes in TTR [30] or chaperone proteins in the liver [31]. It is well known that there is a general increase in the level of protein oxidation with aging [32,33]. It was shown that oxidized TTR was thermodynamically less stable than non-oxidized TTR and had a stronger propensity than non-oxidized TTR to form aggregates and fibrils [30]. Therefore, oxidative modification may be involved in the increased incidence of ATTRwt amyloidosis with aging. In wild-type human *TTR* transgenic mice, young mice (3 months old) did not show cardiac deposition, but half of the 2-year-old mice showed cardiac deposition. After 2 years of expressing a large amount of human TTR, the livers of mice without cardiac deposition showed chaperone gene expression and an activated unfolded protein response, while the livers of mice with cardiac TTR deposition displayed neither [31]. Thus, reduction in the chaperoning capacity of the liver related to aging may result in cardiac deposition.

TTR structural changes, tetramer dissociation and monomer misfolding in vitro are promoted under acidic denaturing conditions [34,35]. However, it is unknown whether acidic conditions such as acidic vesicles are responsible for triggering amyloidogenesis in vivo.

Recently, proteolysis-induced fragmentation of TTR has been proposed as a mechanism facilitating the development of ATTR amyloidosis [36]. Amyloid deposits are classified into type A, which consists of a mixture of cleaved TTR and full-length TTR, and type B, which consists only of full-length TTR. Type A amyloid fibrils are shorter than type B fibrils and have less affinity than type B fibrils for Congo red staining [37,38]. Peptide 49–127 C-terminal fragment is the main component of TTR amyloid fibrils in cardiac deposits, which is further associated with poor clinical prognosis.

There is a clear gender difference in the incidence of ATTRwt with cardiac symptoms, with the majority of cases in men. However, the mechanism of the gender difference is unknown [2,39,40].

#### 3.1.2. Hereditary ATTR Amyloidosis

Hereditary ATTR (ATTRv) amyloidosis is the common autosomal dominant hereditary systemic amyloidosis [11]. In the 1970s, TTR was identified in amyloid deposits in ATTRv amyloidosis patients [41]. Since then, more than 150 variants of the TTR gene have been identified and most of them are amino acid mutations due to a single nucleotide substitution [42].

When a gene mutation occurs in TTR, the TTR tetramer becomes unstable and dissociates into a monomer, which is important for the process of amyloid formation. Actually, several amyloidogenic TTR variants, such as D18G, V30M, L55P, H88R, Y114H, Y116S and V122I TTR, have been demonstrated to exhibit low structural stability in comparison to wild-type TTR [1,43,44,45,46,47]. Dasari et al. showed that wild-type TTR tends to form linear oligomers, while the G53A TTR variant forms annular oligomers with pore-like structures [48]. Frangolho et al. compared the oligomerization processes of wild-type TTR and several TTR variants (V30M, L55P and T119M) and found distinct oligomerization kinetics but a similar oligomerization mechanism [49]. The oligomerization kinetics of the wild-type TTR and TTR variants showed a good correlation with their amyloidogenic potential, and the most amyloidogenic variants aggregated faster (L55P > V30M > wild-type).

In basic research using various TTR mutants, it has been shown that if the TTR is highly unstable due to a mutation, it is likely to cause symptoms of the ocular/central nervous system type or peripheral nerve type, and if it is relatively stable, it likely causes cardiac symptoms [50]. The clinical phenotypes of ATTRv amyloidosis include primary polyneuropathy (V30M), cardiomyopathy (V20I, V122I, L111M, I68L) and mixed phenotype (E89Q, T60A) [51].

The V122I TTR is the most common amyloidogenic mutation worldwide, producing familial cardiomyopathy primarily in individuals of African descent [52].

The S52P variant causes aggressive, highly penetrant systemic amyloidosis [53]. Proteolytic cleavage of this variant generates a residue 49–127 fragment of TTR that rapidly and completely self-aggregates into amyloid fibrils under the condition of physiological fluid agitation [54]. The proteolysis/fibrillation pathway is common to some amyloidogenic variants of TTR and requires the action of mechanical forces provided by the shear stress of physiological fluid flow. The mechanism may be important in the heart, where shear stress is greatest, and the fragment is particularly abundant in cardiac amyloid [55].

V30M TTR is susceptible to plasmin-mediated proteolysis [56]. Bezerra et al. found that SerpinA1, a serine protease inhibitor, inhibited plasmin-mediated V30M TTR proteolysis in vitro [57]. In addition, down-regulation of SerpinA1 increased TTR plasma levels in V30M TTR transgenic mice and cardiac amyloid deposition. Interestingly, the presence of TTR fragments was observed in the heart but not in other tissues following SerpinA1 knockdown.

Conversely, some TTR-stabilizing mutations (R104H, A108V and T119M) have been reported [58,59]. The stabilizing effect of the T119M variant has been investigated in detail [60,61,62]. In a Danish population cohort, it was suggested that carriers of T119M may be protected against cerebrovascular disease and have a longer lifespan [63]. On the other hand, there was no association between the T119M genotype and risk of vascular disease or death in a prospective UK cohort study [64].

#### 3.1.3. Cardiac Deposition of ATTR

Misumi et al. found that amyloid fibers are first formed in the basement membrane of cardiomyocytes and vascular smooth muscle cells and that an increase in basement membrane components and an increase in ATTR deposition occurred in parallel in patients with ATTRv amyloidosis [65]. They also found that expression of basement membrane components was induced by synthetic TTR aggregates in vascular smooth muscle cells and this may contribute to further amyloid deposition.

#### 3.1.4. Toxicity of ATTR to Cardiomyocytes

Direct toxic effects of amyloid on cardiomyocytes have been reported. When cardiac AC16 cells were treated with recombinant TTR variants (V122I, V30M, V20I, L111M and T119M) for 24 h, amyloidogenic variants showed cytotoxicity in a concentration-dependent manner, but the non-amyloidogenic T119M variant did not [66]. However, it is unclear whether toxicity varies among different amyloid fibrils, cardiotropic or non-cardiotropic ones. Manral et al. also demonstrated that an amyloidogenic TTR variant, V122I, generates superoxide species and activates caspase 3/7 in the AC16 human cardiomyocyte cell line, whereas a non-amyloidogenic variant, T119M, does not [67]. Sartiani et al. reported that recombinant wild-type TTR oligomers and aggregates bind to the cell surface and oligomers are internalized into HL-1 cardiac muscle cells. TTR showed significant cytotoxicity and caused mitochondrial dysfunction in HL-1 cells, whereas T_4_-stabilized wild-type TTR did not. TTR oligomers and fibrils also altered calcium cycling and electrophysiological properties of adult mouse left ventricular myocytes [68]. Furthermore, Hein et al. demonstrated that the plasma of healthy individuals or patients with hereditary transthyretin amyloidosis with polyneuropathy promotes hypertrophy of neonatal rat cardiomyocytes induced by phenylephrine, while the plasma of patients with hereditary transthyretin cardiac amyloidosis or wild-type ATTR cardiac amyloidosis attenuated the hypertrophic response in vitro. They also reported that an attenuated cardiomyocyte hypertrophic response after stimulation with patients’ plasma in vitro is an independent risk factor for adverse cardiac events (death, transplantation and/or cardiac decompensation) in ATTR patients [69].

#### 3.1.5. Effects of ATTR on Endothelial Cells

Focusing on the fact that patients with familial amyloidosis polyneuropathy have more thrombotic obstruction of the hepatic artery after liver transplantation [70], Nunes et al. investigated the effects of TTR on vascular endothelial cells. They reported that recombinant V30M TTR tetramers reduced the expression of some pro-angiogenic genes, inhibited cell migration and induced apoptosis in HUVECs [71].

### 3.2. AL Amyloidosis

Amyloid fibrils of AL amyloidosis are derived from monoclonal immunoglobulin light chains (LC) produced by plasma cells. LC are 22–23 kDa proteins consisting of two β-sheets-rich regions: the variable domain (V_L_) and the constant domain (C_L_) [72]. The V_L_ is characterized by high sequence variability due to gene recombination and somatic hypermutation. The C_L_ exhibits limited sequence variation within each of the two light chains isotypes κ and λ. Both λ and κ LC form homodimers via disulfide bond [73]. The majority (approximately 75%) of all monoclonal amyloidogenic LC are λ isotype [13,74]. Some groups have shown unfolding of the LC structure in amyloid fibrils from AL amyloidosis patients using cryo-electron microscopy [75,76,77]. Kazman et al. revealed a multi-step mechanism of LC structual transitions required for fibril formation [78]. The process begins with partial unfolding of V_L_ and forming small amounts of dimers. This is a prerequisite for forming an aggregate of oligomers that are the precursors of fibrils. During oligomerization, rearrangement of the hydrophobic core of the LC domain leads to changes in solvent accessibility and rigidity. Structural transitions from an anti-parallel to a parallel β-sheet secondary structure occur in the oligomers prior to amyloid formation [78]. Kazman et al. also revealed that the combination of proteolytic cleavage and the destabilizing mutation cause structural changes that make the LC pathogenic [79].

Approximately 50–70% of patients with AL amyloidosis have some degree of cardiac involvement [1]. The severity of cardiac involvement remains a major contributor to prognosis.

#### 3.2.1. Toxicity of LC on Cardiac Cells

Liao et al. demonstrated that infusion of LC obtained from AL amyloidosis patients with severe cardiac involvement impaired ventricular relaxation with preservation of contractile function in the isolated mouse heart [80].

Several studies have shown that soluble cardiotropic LC are internalized into cardiomyocytes and cardiac fibroblasts and interact with intracellular proteins, resulting in oxidative stress, mitochondrial ultrastructural changes and apoptosis activation [81,82,83]. Brenner et al. and Shi et al. reported that LC isolated from AL amyloidosis patients with cardiac involvement activated p38 MAPK and induced oxidative stress, contractile dysfunction and apoptosis in isolated adult rat cardiomyocytes [84,85]. McWilliams-Koeppen et al. reported that AL amyloid fibrils composed of recombinant λ6 light chain variable domains decrease NAD(P)H-dependent oxidoreductase without significant cell death in AC10 human ventricular cardiomyocytes. The presence of amyloid fibrils did not affect ATP levels; however, oxygen consumption was increased and reactive oxygen species were detected [86]. Jordan et al. found that exposure to AL fibrils induced changes in the pathways associated with immune response and extracellular matrix components and upregulation of innate immune-associated transcripts (chemokines, cytokines and complement) in cultured cardiomyocytes [87].

#### 3.2.2. Extracellular Matrix Proteolysis in AL Cardiomyopathy

Biolo et al. reported that patients with AL amyloidosis had significantly higher serum MMP9 and TIMP-1 levels than those in patients with ATTR amyloidosis with the same level of left ventricular hypertrophy, and subendocardial myocardial biopsy samples also showed increased expression of MMP9 and TIMP1 [88]. Tanaka et al. also reported that circulating levels of MMPs and TIMPs in AL amyloidosis patients with cardiomyopathy were higher than those in ATTR amyloidosis patients with cardiomyopathy. This suggests significant activation of extracellular matrix proteolysis that interferes with cell–cell coupling and disrupts cellular integrity in AL cardiomyopathy.

## 4. Treatment for Amyloidosis

### 4.1. Treatment for ATTR

#### 4.1.1. Disruption of TTR Aggregation

Doxycycline has been reported to eliminate TTR aggregates in ATTR transgenic mice [89]. Some mechanisms, including matrix metalloproteinase inhibition and involvement of extracellular chaperones, such as cluster phosphorus, have been proposed [20,90].

Tauroursodeoxycholic acid (TUDCA) has been reported to inhibit aggregate formation of non-fibrotic TTR and has been shown to be effective in animal studies [89,91]. TUDCA has been used with doxycycline in patients with ATTR cardiac amyloidosis [92,93]. However, its effectiveness has not been established clinically.

CLR01, a lysine-specific molecular tweezer, inhibits the self-assembly and toxicity of different amyloidogenic proteins, including TTR, in vitro by interfering with hydrophobic and electrostatic interactions that play an important role in the aggregation process [94]. CLR01 administration reduced TTR deposits in V30M TTR transgenic mice, a model of ATTRv amyloidosis [95].

Several natural polyphenols have been reported to have potent inhibitory effects on amyloid fibril formation. Curcumin, a natural polyphenol presenting structural similarities with T_4_, binds to TTR and suppresses TTR amyloid fibril formation by generating small non-toxic oligomers, and (-)-epigallocatechin gallate (EGCG), the most abundant catechin in green tea, maintains most of the protein in a non-aggregated soluble form. Both efficiently disaggregate pre-formed TTR amyloid fibrils [96]. In addition, Curcumin or EGCG treatment decreased TTR deposition and levels of endoplasmic reticulum (ER) stress markers in TTR V30M transgenic mice [97,98,99].

#### 4.1.2. Stabilization of TTR Tetramers

TTR normally exists in vivo as a tetramer, and dissociation from a tetramer to monomers is required for TTR to form amyloid fibrils. Since TTR aggregation is very efficient once the misfolded monomer state is reached, small compounds that stabilize the TTR tetramer via binding to T_4_ binding sites have been developed [60,100,101,102,103,104,105,106]. Alhamadsheh et al. reported that some compounds showed V122I TTR cytotoxicity in human cardiomyocytes in vitro [106].

Nonsteroidal anti-inflammatory drugs (NSAIDs) stabilize native TTR tetramers and suppress TTR amyloid fibril formation [107]. Diflunisal binds to the unoccupied T_4_ binding sites in TTR, and therapeutic serum concentrations of diflunisal stabilize serum variant TTR tetramer [108,109]. Orally administered diflunisal significantly increased the serum TTR concentration and stabilized the TTR tetramer structure in ATTRv patients; however, attention should be paid to renal dysfunction and thrombocytopenia associated with difunisal administration [110,111].

AG10 binds to TTR T_4_ binding sites and mimics the stabilizing T119M variant to stabilize the TTR tetramer structure [112]. AG10 is unique in its ability to form hydrogen bonds with the same serine residue at position 117 that stabilizes the T119M TTR. AG10 prevented dissociation of wild-type and V122I TTR in serum samples from ATTR patients with cardiomyopathy [113]. In a phase 2 trial, AG10 was well tolerated and it stabilized TTR and restored low TTR levels to normal levels in all ATTR patients with cardiomyopathy [114]. A phase 3 trial is ongoing (NCT03458130).

Tolcapone, a drug for the treatment of Parkinson’s disease, has been repositioned to treat ATTR amyloidosis [115,116]. Tolcapone specifically binds to wild-type, A25T, V30G, Y114C and V122I TTR and inhibits fibril formation, stabilizes native tetramers in vivo in mice and humans and inhibits TTR cytotoxicity [115]. Furthermore, a phase 2a trial showed the TTR stabilizing ability of tolcapone in all participants including ATTRv patients and healthy volunteers [117].

Tafamidis also binds to TTR T_4_ binding sites and stabilizes TTR tetramers to prevent production of TTR monomers [118]. In the ATTR-ACT clinical trial, tafamidis actually improved all-cause mortality, rates of cardiovascular-related hospitalization, distance for the 6-min walk test and Kansas City Cardiomyopathy Questionnaire overall summary score in patients with TTR amyloid cardiomyopathy. However, it was only effective when heart failure was mild [8]. This suggests that it is necessary to not only suppress ATTR production but also remove the accumulated ATTR for patients with long-term morbidity.

#### 4.1.3. Suppression of TTR Synthesis

Tsuchiya et al. reported that amyloid deposits regressed in ATTRv patients after liver transplantation, which removes the main source of mutant TTR [119].

Treatment with patisiran, an siRNA targeting a conserved sequence in the 3′ untranslated region of *TTR* resulted in an 84% mean reduction of serum TTR at 18 months from baseline [6]. Treatment with inotersen, a 20-base antisense oligonucleotide targeting *TTR* mRNA, resulted in a 77% mean reduction of serum TTR at 15 months [7]. Groothof et al. also reported a case with regression of cardiac bone-tracer uptake, ventricular wall thickness and TTR deposition in abdominal adipose tissue after addition of inotersen or patisiran to tafamidis [120]. These results suggest that the already accumulated TTR is eliminated from the extracellular matrix once a drastic reduction in mutant TTR production is achieved.

Interestingly, Finn et al. achieved a >90% reduction of serum TTR levels following a single systemic administration of CRISPR/Cas9 lipid nanoparticles against *Ttr* in mouse and rat models [121]. Furthermore, Gillmore et al. reported gene editing in hereditary ATTR patients with 0.3 mg/kg CRISPR/Cas9 targeting *TTR* and achieved an 87% reduction of serum TTR levels at day 28 [9].

It is known that knocking out the *TTR* gene in animal models does not show a clear phenotype [122]. However, TTR has various functions, such as proteolytic activity toward apoA-I, neuropeptide Y and amyloid β-peptide, and a neuroprotective role against cerebral ischemia in addition to transporting T_4_ and retinol-binding protein [123,124,125,126]. Therefore, the long-term consequences should be carefully considered.

#### 4.1.4. Removal of TTR Aggregates

Cells have a mechanism to take up and degrade TTR. Misumi et al. showed that NIS3T3 fibroblast cells endocytosed and degraded not only soluble TTR but also aggregated TTR [127]. Furthermore, both fibroblasts and macrophages internalize and clear not only soluble TTR but also aggregated wild-type TTR and V30M TTR when subcutaneously injected into mice over time. In addition, in autopsy heart tissues obtained from familial amyloid polyneuropathy V30M patients containing amyloid deposits, epicardial and endocardial fibroblasts were TTR-positive in immunohistochemistry.

The removal of ATTR with monoclonal antibodies is being investigated [128,129,130,131]. The targets are new epitopes exposed on the molecular surface with a conformational change of TTR during aggregation and antibodies do not bind TTR [132]. ATTR is removed by phagocytic immune cells including macrophages.

#### 4.1.5. Anti-Seeding Therapy

Since the liver is the primary source of TTR production, patients with ATTRv amyloidosis have been treated by liver transplantation to replace the mutant TTR gene with a wild-type gene [133]. In many ATTRv cases, liver transplantation results in an improved prognosis with stabilization or slowing progression of the disease; however, this procedure may be followed by progressive cardiac deposition of wild-type TTR secreted by the new liver [134]. Several studies have shown predominance of wild-type TTR over variant TTR in cardiac amyloid isolated from patients after liver transplantation [135,136]. In addition, type A deposits, consisting of a mixture of cleaved TTR and full-length TTR, are highly capable of recruiting wild-type TTR [37]. Saelices et al. demonstrated that amyloid fibrils extracted from autopsied and explanted hearts of ATTRv patients who had undergone liver transplantation robustly seed wild-type TTR into amyloid fibrils in vitro. Furthermore, they designed a structure-based peptide inhibitor, TabFH2, to prevent this seeding process ex vivo [137,138].

### 4.2. Treatment for AL Amyloidosis

#### 4.2.1. Elimination of Light Chain Sources

The basic treatment for AL amyloidosis is to reduce the free LC, which are the source of amyloid, by methods such as chemotherapy and autologous stem cell transplantation [5,139,140,141,142].

Daratumumab is a monoclonal antibody that binds to CD38, an antigen highly expressed on the surface of myeloma plasma cells. The plasma cell clone in AL amyloidosis also expresses CD38, and a high level of CD38 expression is associated with poor survival in patients with AL amyloidosis [143]. Daratumumab induces myeloma plasma cell death through complement-dependent cytotoxicity, antibody-dependent cell-mediated cytotoxicity, antibody-dependent cellular phagocytosis and direct cellular apoptosis [144,145].

Although AL amyloid regression has been reported in some cases after chemotherapy and/or stem cell transplantation [146,147,148,149], treatments that promote the removal of amyloid deposited in organs have not yet been clinically established.

#### 4.2.2. Disruption of Light Chain Aggregation

Ward et al. reported that doxycycline reduced fibril formation in a transgenic mouse model of AL amyloidosis [150]. They also showed that doxycycline suppresses fibril formation and aggregation of recombinant amyloidogenic LC [150]. Furthermore, doxycycline has been shown to have beneficial properties in retrospective studies [151,152]. However, a placebo-controlled study is needed [153].

#### 4.2.3. Removal of Amyloid Deposits

Treatment with monoclonal antibodies against amyloid deposits is being investigated [154,155,156].

NEOD001 is a humanized form of murine monoclonal antibody 2A4, which binds to an epitope on the misfolded light chain protein but does not bind to the native conformation [156,157]. A phase 2b study in previously treated patients with a hematologic response who had persistent cardiac dysfunction (PRONTO NCT02632786) failed and the development of NEOD001 was discontinued [158]. CAEL-101 is also a monoclonal antibody that binds to the conformational epitope present on human light chain amyloid fibrils [159]. CAEL-101 was evaluated in a phase 1a/b trial in patients with relapsed/refractory AL amyloidosis [160]. Of the ten patients who received CAEL-101, nine had improvement in global longitudinal strain (GLS). Improvement in GLS correlated with a reduction of NT-pro BNP [161].

Serum amyloid P component (SAP) is a glycoprotein that binds to various types of amyloid fibrils [162]. Bodin et al. showed that anti-human SAP antibodies activated macrophage-mediated phagocytosis of the human SAP containing amyloid deposits in mice [154]. A phase 2 trial in patients with cardiac amyloidosis (NCT03044353) failed to show any improvement in cardiac amyloid burden after treatment with an anti-SAP antibody (dezamizumab).

#### 4.2.4. Stabilization of Amyloidogenic LC

Morgan et al. identified small molecules that kinetically stabilize the native dimeric structure of full-length LC by binding at the V-domain–V-domain interface in full-length LC. Stabilization of full-length light chain reduces the rate at which LC undergo conformational excursions leading to either aggregation of LC or aberrant endoproteolysis and aggregation of light chain fragments [163].

## 5. Summary

**Figure 1:**
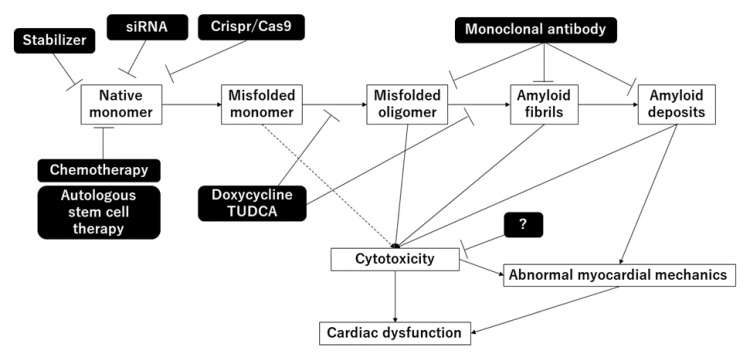
Mechanisms of amyloidosis and treatments.

In this review, we summarized the mechanisms and treatments of cardiac amyloidosis. The mechanisms are summarized in Figure 1. In recent years, the development of treatments that suppress the production of abnormal proteins has progressed, and improvement in prognosis is expected. However, there are still unknown mechanisms and it is expected that clarification of these mechanisms will lead to the development of further treatment methods.

## Figures and Tables

**Table 1:** Classification of cardiac amyloidosis.

	Precursor Protein	Underlying Condition	Cardiac Involvement	Treatment
ATTRwt	Wild-type transthyretin	Aging	++	TTR tetramer stabilizer
ATTRv	Abnormal transthyretin	*TTR* gene variant	+	Liver transplantation Transthyretin tetramer stabilizer siRNA/antisense oligomer
AL	Immunoglobulin light chain	Plasma cell abnormality	++	Chemotherapy Autologous stem cell transplantation

+, ++, Cardiac involvement (upper line).

## Abbreviations

AA	amyloid A
AL	amyloid light-chain
ATTR amyloidosis	transthyretin amyloidosis
ATTRv	hereditary ATTR amyloidosis
ATTRwt	wild-type ATTR amyloidosis
siRNA	small interfering RNA
TTR	transthyretin
